# Somatostatin-SSTR3-GSK3 modulates human T-cell responses by inhibiting OXPHOS

**DOI:** 10.3389/fimmu.2024.1322670

**Published:** 2024-02-15

**Authors:** Bo Zhang, Huiru Feng, Hui Lin, Rui Li

**Affiliations:** ^1^ Institute of Neuroscience and Fujian Key Laboratory of Molecular Neurology, Fujian Medical University, Fuzhou, China; ^2^ Institute of Clinical Research, Fujian Medical University, Fuzhou, China; ^3^ Institute of Immunotherapy and Department of Neurology of First Affiliated Hospital, Fujian Medical University, Fuzhou, China

**Keywords:** somatostatin (SST), T cells, somatostatin receptor 3 (SSTR3), mitochondrial respiration and glycogen synthase kinase-3 (GSK3), metabolism

## Abstract

**Introduction:**

Somatostatin (SST) is a peptide hormone primarily synthesized in the digestive and nervous systems. While its impact on the endocrine system is well-established, accumulating evidence suggests a crucial role for SST and its analogues in modulating immune responses. Despite this, the precise mechanism through which SST regulates T cells has remained largely unknown.

**Methods:**

To elucidate the impact of SST on human T cells, we conducted a series of experiments involving cell culture assays, molecular analyses, and metabolic profiling. Human T cells were treated with SST, and various parameters including proliferation, cytokine production, and metabolic activities were assessed. Additionally, we employed pharmacological inhibitors and genetic manipulations to dissect the signaling pathways mediating SST's effects on T cells.

**Results:**

We showed that SST diminishes T-cell proliferation by influencing IL-2 production and T-cell mitochondrial respiration, while having no discernible impact on TCR-induced glycolysis. Our findings also identified that the regulatory influence of SST on T-cell responses and metabolism is contingent on its receptor, SSTR3. Moreover, we demonstrated that SST governs T-cell responses and metabolism by acting through the T-cell metabolic checkpoint GSK3.

**Discussion:**

Our study provides novel insights into the immunoregulatory function of SST in human T cells, highlighting the complex interplay between hormonal signaling and immune regulation. Understanding the molecular mechanisms underlying SST's effects on T cells may offer therapeutic opportunities for manipulating immune responses in various pathological conditions.

## Highlights

- SST decreases T-cell responses and mitochondrial respiration in human.
- SST regulates T-cell responses through SSTR3.
- SSTR3 reduce T-cell responses and mitochondrial respiration through GSK3.

## Introduction

Somatostatin (SST), a peptide hormone intricately synthesized within the digestive and nervous systems, emerges as a pivotal inhibitory regulator in the complex endocrine cascade (1). Serving as a master orchestrator, the primary function of SST is to suppress multiple hormones such as growth hormone and gastrointestinal hormones (2). There are five known subtypes of SST receptors: SSTR1-5, each exhibiting a unique distribution and affinity for SST (2). SSTRs are expressed by many tissues, such as brain and gut, as well as most lymphatic tissues, including gut-associated lymphatic tissue, spleen and thymus (3). The activation of somatostatin receptors by somatostatin or its analogs initiates intracellular signaling cascades, leading to diverse physiological effects, including the inhibition of cell proliferation, and neurotransmission. The distinct distribution and functions of somatostatin receptors contribute to the pleiotropic regulatory role of somatostatin in maintaining homeostasis across multiple organ systems (2).

The dysregulation of SST-SSTRs has been implicated in diseases such as Alzheimers diseases. It has been reported that memory loss in patients with Alzheimers Disease (AD) may have been derived from deficits in somatostatin function (4). Of note, due to its inhibitory function, the analogue of SST has been developed to treat many diseases including Acromegaly, Cushings disease, and gastrointestinal disorders. Deciphering somatostatin receptor signaling is crucial for unraveling the complexities of somatostatins physiological actions and exploring potential therapeutic applications in various medical conditions.

A growing body of evidence underscores the potential pivotal role of SST and its analogues in modulating immune responses (5–13). For example, using (3) H thymidine incorporation assay, previous study showed that SST could reduce T-cell proliferation within peripheral blood mononuclear cells (PBMC) in response to mitogen such as Phytohaemagglutinin (PHA) and Concanavalin A (ConA) (8). However, in the context of PBMC, it is hard to determine whether this is the direct effect of SST on T cells or some indirect actions through other immune cells.

In the realm of disease, experimental investigations have revealed that ingested SST can ameliorate conditions like experimental autoimmune encephalomyelitis, a T-cell-mediated animal model for neuroinflammation (11). Notably, this amelioration is associated with a significant reduction in Th1 and Th17 responses (11). Furthermore, the administration of SST analogs has demonstrated efficacy in diminishing immune-mediated arthritis (12). Consequently, there is growing interest in exploring the therapeutic potential of SST and its analogs for inflammatory diseases, such as inflammatory bowel diseases and rheumatoid arthritis, despite the mechanism underlying the regulatory effect of SST on T-cell remain largely unknown.

In our current study, we embarked on elucidating the molecular underpinnings of SSTs immune regulatory role in human T cells. Our findings underscore that SST exerts a suppressive influence on T-cell responses, marked by a preferential reduction in mitochondrial respiration, rather than impacting glycolysis. Additionally, we unveil that SST modulates T-cell responses and metabolism through the SSTR3-induced activation of GSK3. This study contributes novel insights into the immune regulatory functions of SST, paving the way for a more comprehensive understanding of its therapeutic potential.

## Materials and methods

### Ethics, consent and permissions

All subjects provided written informed consent as approved by the Fujian Medical University ethics review board (Ref. 2023-70 and Ref. 2023-71). Healthy subjects were recruited from Fujian Medical University. We excluded donors with known history or presence of chronic diseases, autoimmune diseases, infectious diseases, and cancer. The age range of the donors included was 25~45 at the time of blood draw.

### Cell culture

Human peripheral blood mononuclear cells (PBMC) were separated by density centrifugation using Ficoll (GE health care). CD3^+^ T cells, CD4^+^ T cells, CD8^+^ T cells, naive T cell subsets and memory T cells, were all isolated using negative selection (Miltenyi biotec) with confirmed purities of > 97%. Cells were then labelled with Carboxyfluorescein succinimidyl ester (CFSE, ThermoFisher) to track cell proliferation. All cells were cultured in serum-free X-vivo 10 media (Lonza). T cells were plated in U bottom 96 well plate at 1x105 cells/well in a total volume 200ul of medium. Polyclonal activation beads (αCD2, αCD3 and αCD28, Bead to Cell Ratio: 1:2, Miltenyi) were used to stimulate T cells for 4 days. Reagents for cell culture include recombinant somatostatin (100nM, S9129, Sigma-Aldrich); SSTR3i (MK-4256, 2nM, AdooQ Bioscience); GSK3 inhibitor (CHIR 99021, 20nM, TOCRIS); IL-2 (100U/ml, ThermoFisher.).

### Flow cytometry staining

Cells were first washed once with PBS. Live/Dead Aqua (0.5ul per sample, ThermoFisher) staining was then performed at room temperature. After two washes, cells were then incubated with surface antibody cocktails. To assess intracellular cytokines, PMA (10ng/ml, Sigma-Aldrich), Ionomycin (500ng/ml, Sigma-Aldrich) and Golgi stop (BD bioscience) were added to cells 4 hrs before staining. Cells were then stained with live/dead aqua marker, following which cell-surface marker staining was performed. Cells were then fixed and permeabilized using fixation/permeabilization buffer (BD bioscience). cytokine antibodies were added and incubated for 30 min. Samples were then washed twice and analyzed by FACSVerse (BD Bioscience). Antibodies used for flow cytometry staining in this study include: CD3-PerCp Cy5.5 (SK7, BD Bioscience), CD4-APC-Cy7 (RPA-T4, BD Bioscience), CD8-PE-Cy7 (RPA-T8, BD Bioscience), SSTR3-Alexa 647 (FAB7018R, R&D system), CD25-PE (BC95, BD Bioscience), CD69-APC (FN50, BD Bioscience), GM-CSF-BV421 (BVD2-21C11, BD Bioscience), IL-17A-PE (N49-653, BD Bioscience), IL-2-PE (5344.111, BD Bioscience) and IFNγ-APC (B27, BD Bioscience).

### ELISA

Cytokine in the culture supernatants were measured by ELISA (IFNγ, GM-CSF and IL-10 BD bioscience, and IL-17A, ebioscience) following the manufacturers protocols. Briefly, ELISA plates (Nunc MaxiSorp™ flat bottom) were coated with capture antibodies overnight. After 3 washes with washing buffer (0.05% Tween 20 in PBS), plates were blocked by 10% FCS for 1 hour. Plates were then washed 3 times with washing buffer. Samples with proper dilution were then added to the plate and incubated for two hours. Following 5 washes, detection antibodies were added and incubated for 1 hour at room temperature. The plate was carefully washed with ELISA washing buffer. The color of the plate was developed by TMB (BD bioscience) and the reaction was stopped by 0.01N H_2_SO_4_. The phosphorylation of GSK3 (inactive form) and total GSK3 are measured using InstantOne ELISA kit (85-86173-11, ThermoFisher) following manufacturers protocols. The plates were read by a ThermoFisher microplate reader (Multiskan™ FC Microplate Photometer).

### Seahorse assay

T-cell mitochondrial respiration and glycolysis were detected by XF Mitochondria stress test kit and Glycolytic rate assay kit (Agilent) following the manufacturers protocols. For MitoStress test, T cells were cultured under various conditions for 24hrs. For glycolytic rate assay, T cells were cultured under various conditions for 15min. Cells were then harvested, counted and then re-plated in Cell-tak (22.4ug/ml, Corning) coated XF96 microplates with same density of live cells (5x10 (5)/well) across different conditions. The plate was then centrifuged at 200g for 1 min to accelerate the attachment of cells to the plate. The plate was then transferred to a 37°C incubator without supplement of CO_2_ for 60min before analyzed by Seahorse XF96 analyzer. Key component for the assay: XF media (non-buffered DMEM+10mM Glycose, 4mM L-glutamine, and 2mM sodium pyruvate), Oligomycin: 1uM, FCCP: 1uM, Rotenone/AA: 1uM and 2-DG: 200mM.

### Real-time PCR

RNA was extracted from purified human primary T cells using RNeasy-mini kit (Qiagen). Reversed Transcriptional PCR (Transcriptor First Strand cDNA Synthesis Kit, 04379012001, Roche) was performed generate the cDNA. The expression of somatostatin receptors was measured by real-time PCR (StepOne™ real-time PCR, ThermoFisher) and normalized based on actin. The primer used in this study include: sstr1 (Hs00265617_s1, ThermoFisher), sstr2 (Hs00265624_s1, ThermoFisher), sstr3 (Hs00265633_s1, ThermoFisher), sstr4 (Hs01566620_s1, ThermoFisher), sstr5 (Hs00990407_s1, ThermoFisher), and β-actin (Hs99999903_m1, ThermoFisher).

### Transfection

Total T cells were transfected using the Amaxa Nucleofection system and the Amaxa Nucleofector kit for human T cells according to the manufacturers instructions. Briefly, siRNA-treated T cells (5 × 10^6^/100 μl) were mixed with 1 μg of the indicated siRNA. Samples were then transferred to cuvets. After transfection, cells were rested in x-vivo medium for 24hrs; On day 2, cells were washed, counted, and resuspended in fresh medium and stimulated with anti-CD2/CD3/CD28 microbeads. SSTR3 and GSK3 total protein levels were assessed by flow cytometry and ELISA respectively. The siRNA used in this study include: SSTR3 siRNA (ID: 289278; 41631 and 41720); GSK3beta siRNA (ID: 144880; 144881 and 144882); control siRNA (Cat. No. 4390843).

### Statistics

All values are expressed as either individual dot or means ± SD, and p-values were assessed as appropriate by either two-tailed paired students t-test or repeat measure two-way ANOVA with two-stage step-up method of Benjamini, Krieger and Yekutieli to correct for multiple comparison by controlling the False Discovery Rate using Graphpad Prism version 9. ns: not significant, *p<0.05; **p<0.01; ***p<0.001 and ***p<0.001.

## Results

### SST decreases T-cell activation and proliferation

**Figure 1:**
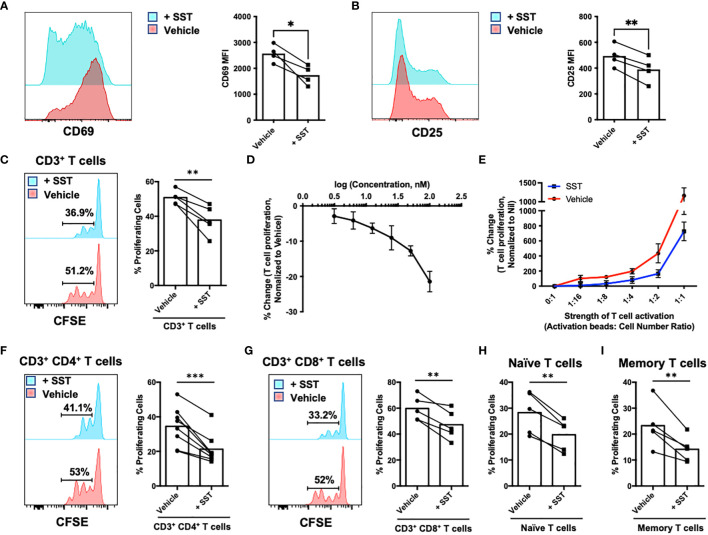
SST down-regulates T-cell proliferation. **(A, B)** Purified human primary T cells were treated either with vehicle or SST for 30min and then stimulated with T-cell activation beads (αCD2, αCD3 and αCD28) for 6hrs. CD25 and CD69 expression was measured by FACS (n=4). Two-tailed, paired Students t test. *p<0.05. **(C)** Human purified T cells were labelled with Carboxyfluorescein succinimidyl ester (CFSE) and were pre-treated with SST (100nM) for 30min. After that, T-cell activation beads were used to stimulate T cells at 1:2 ratio (beads: cells) for 4 days. T-cell proliferation were measured by the dilution of CFSE and quantified by flow cytometry. Compared to Vehicle condition, SST decreases T-cell proliferation (n=5). **(D)** Dose titration of SST on T-cell proliferation (n=3). SST down-regulates T-cell proliferation in a dose dependent manner. **(E)** Dose titration of activation beads to modulate the strength of T-cell activation. SST dampens T-cell proliferation in across range of T-cell activation (n=3). **(F-I)** Human purified T-cell subsets were labelled with CFSE and were pre-treated with SST (100nM) for 30min. After that, T-cell activation beads were used to stimulate T cells at 1:2 ratio (beads: cells) for 4 days. The proliferation of different T-cell subsets was measured by the dilution of CFSE and quantified by flow cytometry. SST equally decreases CD4^+^ **(F)**, n=8) and CD8^+^ **(G)**, n=5) T-cell proliferation. SST also reduced both naive **(H)**, n=5) and memory **(I)**, n=5) T-cell responses. *Vehicle:* ddH_2_O. Data is presented as individual dots or mean±SD, Two-tailed, paired Students t test. *p<0.05, **p<0.01 and ***p<0.001.

To investigate the potential direct impact of somatostatin (SST) on T-cell activation and proliferative response, we first isolated CD3^+^ T cells from human peripheral blood and labelled them with CFSE to track T-cell proliferation. T cells were either pretreated with SST or vehicle for 30min and then stimulated with αCD2, αCD3 and αCD28 beads. Early T-cell activation markers CD69 and CD25 were measured at 12hrs, and T-cell proliferation were measured at day 4. While pre-treatment of SST does not change human T-cell survival (**Supplementary Figure 1A**), it significantly decreased T-cell activation (**Figures 1A, B**) and proliferation in a dose-dependent manner (**Figures 1C, D**) and this effect of SST can be detected at various strength of T-cell stimulation (**Figure 1E**). In addition, using purified CD4^+^ or CD8^+^ T cells, we found that SST reduced both CD4^+^ and CD8^+^ T-cell proliferation (**Figures 1F, G**). Furthermore, there is no preferential effect of SST on either naive or memory T cells (**Figures 1H, I**). And SST does not seem to affect TCR signaling pathway as measured by pLck and pZap70 (**Supplementary Figures 1B, C**). Together, these data suggested SST can decreases T-cell activation and proliferation in human.

### SST reduces T-cell cytokine production

**Figure 2:**
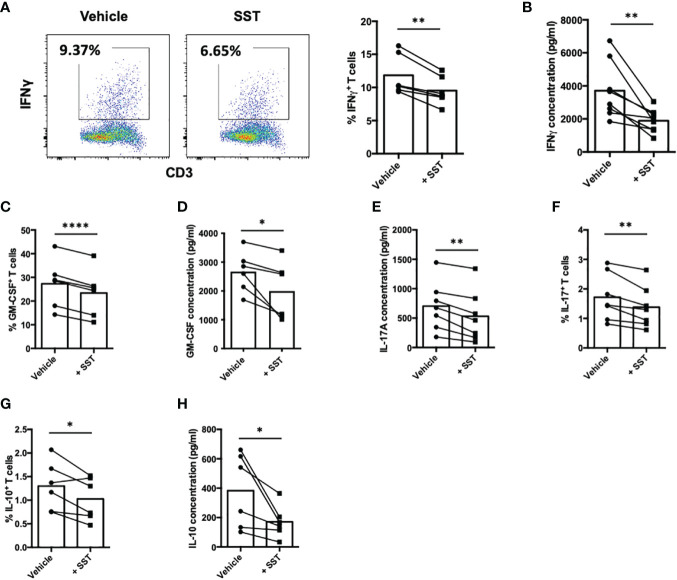
SST modulates T-cell cytokine responses. **(A-H)** Human purified T cells were labelled with CFSE and were pre-treated with SST (100nM) for 30min. After that, T-cell activation beads were used to stimulate T cells at 1:2 ratio (beads: cells) for 4 days. Cytokines from T cells were measured by FACS intracellular staining and ELISA. Compared to Vehicle condition, SST decreases IFNγ **(A, B)**, n=8), GM-CSF **(C, D)**, n=6), IL-17 **(E, F)**, n=7) and IL-10 **(G, H)**, n=6) production by T cells. *Vehicle:* ddH_2_O. Data is presented as individual dots, Two-tailed, paired Students t test. *p<0.05; **p<0.01; and ****p<0.0001.

Cytokines produced by T cells play a crucial role as immune mediators in the modulation of local inflammation. In our subsequent investigations, we sought to determine whether somatostatin (SST) could exert an influence on T-cell cytokine expression. Employing a similar culture system as described earlier, we evaluated T-cell cytokine expression through both intracellular cytokine staining (ICS) and enzyme-linked immunosorbent assay (ELISA). These cytokines include: IFNγ, GM-CSF, IL-17, IL-10 and IL-2. Compared to the vehicle control, SST significantly decreased IFNγ expression by T cells (**Figures 2A, B**). SST also moderately modulate other cytokines from T cells (**Figures 2C–H**).

**Figure 3:**
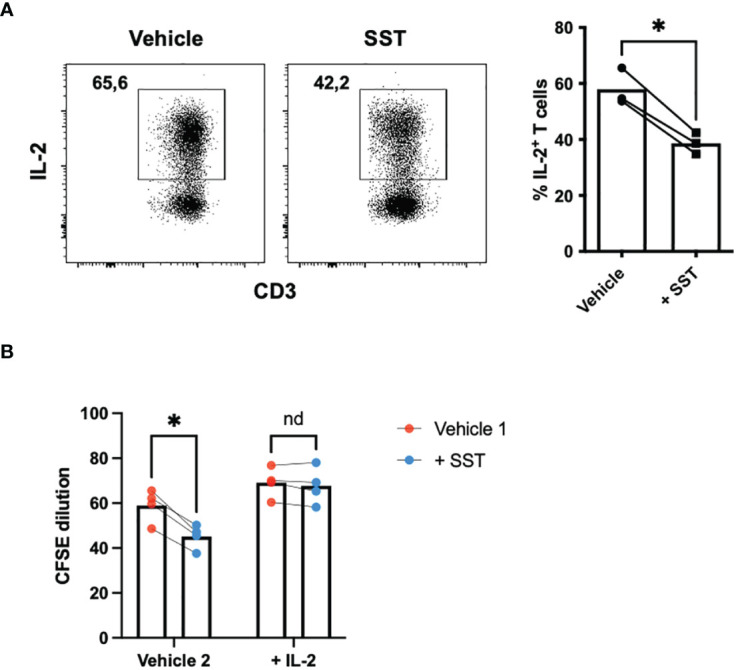
SST reduces T-cell proliferation by down-regulation of IL-2. **(A)** Purified human primary T cells were treated either with vehicle or SST for 30min and then stimulated with T-cell activation beads (αCD2, αCD3 and αCD28) for 4 days. IL-2 expression was measured by ICS (n=3). Two-tailed, paired Students t test. *p<0.05. **(B)** CFSE labeled human primary T cells were treated either with vehicle or SST for 30min and then stimulated with T-cell activation beads (αCD2, αCD3 and αCD28) in presence or absence of recombinant IL-2 for 4 days. CFSE dilution was quantified by flow cytometry (n=4). Vehicle 1: ddH_2_O. Vehicle 2: PBS. Data is presented as individual dots. Repeat measure two-way ANOVA. nd, not different, *p<0.05.

Of particular significance is IL-2, a pivotal cytokine known for its ability to promote T-cell proliferation. Our data indicated that SST may downregulate IL-2 expression by T cells (**Figure 3A**). Prompted by this observation, we sought to investigate whether the suppressive effect of SST on T-cell proliferation could be attributed to the reduction of IL-2. To test that, we added recombinant IL-2 together with SST, we found that IL-2 could reverse the effect of SST on T-cell proliferation (**Figure 3B**), suggesting that SST may modulate T-cell proliferation by decreasing T-cell IL-2 expression.

### SST modulates T-cell metabolism in human

**Figure 4:**
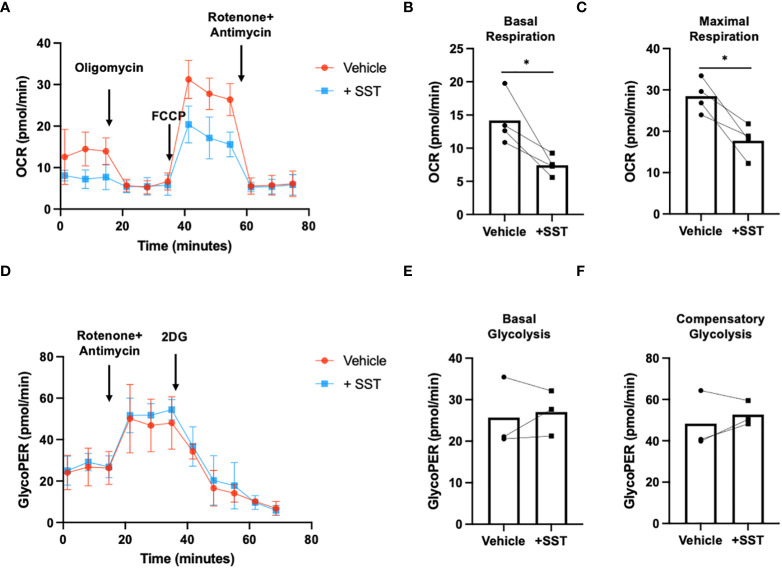
SST modulates T-cell mitochondrial respiration but not glycolysis. **(A-C)** Human purified T cells were pre-treated with SST (100nM) for 30min. After that, T-cell activation beads were used to stimulate T cells at 1:2 ratio (beads: cells) for 24hrs. XF Mitochondria stress test was used to detect T-cell mitochondrial respiration. Compared to Vehicle condition, SST decreases both T-cell basal respiration **(B)**, n=4) and maximal respiration **(C)**, n=4). **(D-F)** Human purified T cells were pre-treated with SST (100nM) for 30min. After that, T-cell activation beads were used to stimulate T cells at 1:2 ratio (beads: cells) for 15min. XF Mitochondria stress test kit was used to detect T-cell mitochondrial respiration. Glycolytic rate assay was used to detect T-cell glycolysis. SST does not change T-cell glycolysis **(E, F)**, n=3). *Vehicle:* ddH_2_O. Data is presented as individual dots or mean±SD, Two-tailed, paired Students t test. *p<0.05.

Research has highlighted the pivotal role of immunometabolism in orchestrating T-cell responses (both proliferation and effector function) (14). We explored whether SST may modulate T-cell metabolism, which may in turn influence T-cell responses. We employed seahorse assays to quantify T-cell metabolism (**Figure 4A**).

In comparison to the vehicle control, treatment with SST emerged as a significant modulator of T-cell mitochondrial respiration, affecting both basal respiration (**Figure 4B**) and maximal respiration (**Figure 4C**). Notably, SST did not appear to change T-cell glycolysis (**Figures 4D–F**). This collectively suggested that SST preferentially impact mitochondrial mediated T-cell metabolism in humans, which is associated with decreased T-cell responses.

### SST regulates T-cell responses through SSTR3

**Figure 5:**
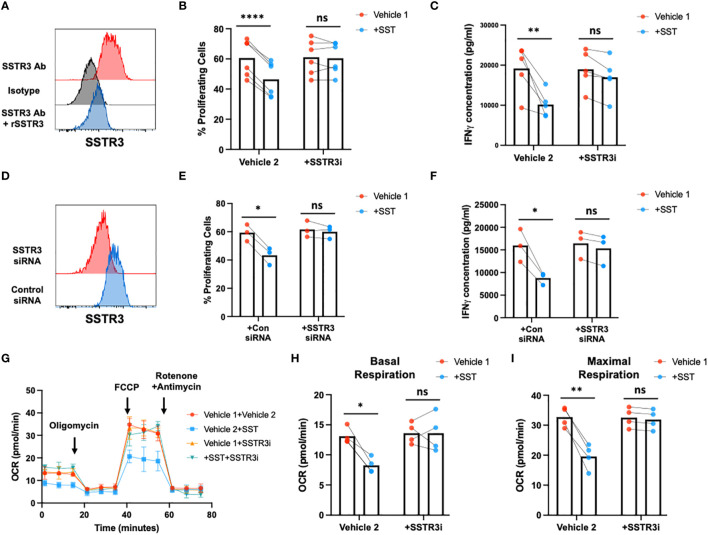
SST modulates T-cell responses through SSTR3. **(A)** The expression of SSTR3 on fresh isolated T cells were measured by flow cytometry (number on the graph represent mean fluorescence intensity, MFI). T cells express SSTR3 at protein level (A, n=3). **(B, C)** Human purified T cells were labelled with CFSE and were pre-treated with SSTR3 inhibitor (SSTRi, MK-4256, 2nM) for 30min. Cells were then incubated with SST (100nM) for 30min. After that, T-cell activation beads were used to stimulate T cells at 1:2 ratio (beads: cells) for 4 days. T-cell proliferation were measured by the dilution of CFSE and quantified by flow cytometry. IFNγ from T cells was measured by ELISA. The blockade of SSTR3 inhibits the effect of SST on T-cell proliferation **(B)**, n=6) and IFNγ production **(C)**, n=6). **(D-F)** CFSE labeled human primary T cells were transfected with either control siRNA or SSTR3 siRNA mix. On day 2, the expression of SSTR3 were measured by FACS **(D)** After transfection, cells were pre-treated with SST for 30min and then stimulated with T-cell activation beads (αCD2, αCD3 and αCD28) for 4 days. CFSE dilution **(E)** and IFNg expression **(F)** was measured by flow cytometry and ELISA respectively (n=3). **(G-I)** Human purified T cells were pre-treated with SSTR3 inhibitor (SSTRi, MK-4256, 2nM) for 30min. Cells were then incubated with SST (100nM) for 30min. After that, T-cell activation beads were used to stimulate T cells at 1:2 ratio (beads: cells) for 24hrs. XF Mitochondria stress test was used to detect T-cell mitochondrial respiration. The blockade of SSTR3 reverses the effect of SST on T-cell mitochondrial respiration **(G-I)**, n=4). *Vehicle 1:* ddH_2_O. *Vehicle 2:* DMSO. Data is presented as individual dots or mean±SD. Repeat measure two-way ANOVA, Two-stage step-up method of Benjamini, Krieger and Yekutieli to correct for multiple comparison by controlling the False Discovery Rate. ns, not significant, *p<0.05; **p<0.01; and ***p<0.001.

Subsequently, we sought to unravel how SST may shape T-cell responses. In the human system, five distinct receptors for SST have been identified (SSTR1~5) (15). To delve into this, we initially assessed the expression of SST receptors in freshly isolated T cells using real-time PCR (**Supplementary Figure 2A**). Our results revealed that among the known receptors, SSTR3 emerged as the predominant SST receptor expressed by T cells (**Supplementary Figure 2A**). Notably, the expression of SSTR3 remained consistent across various T-cell subsets [**Supplementary Figure 2B** (CD4^+^ T-cell subsets) and C (CD8^+^ T-cell subsets)]. We further confirmed SSTR3 expression at protein level on T cells using flow cytometry (**Figure 5A**). Intriguingly, the activation of T cells appeared to downregulate SSTR3 expression on these cells (**Supplementary Figures 2D, E**).

To test whether SST regulate T-cell responses through SSTR3, we blocked SSTR3 using MK-4256 or siRNAs. Remarkably, the inhibition of SSTR3 eliminated the observed impact of SST on both T-cell proliferation and IFN-γ expression (**Figures 5B–F**). Furthermore, this inhibition of SSTR3 effectively reversed the influence of SST on T-cell mitochondrial respiration (**Figures 5G–I**). Collectively, these findings strongly suggest that SST modulates T-cell responses through SSTR3 and shed light on the SSTR3-driven mechanisms underlying the regulatory effects of somatostatin on T-cell behavior.

### SST decreased T-cell responses through SSTR3 induced GSK3

Earlier research has elucidated that the activation of glycogen synthase kinase 3 (GSK3) mediated by somatostatin receptor 3 (SSTR3) constitutes a crucial downstream signaling pathway underlying the effects of somatostatin (SST) (16). Moreover, GSK3, recognized as a metabolic checkpoint regulator, plays a significant role as a negative modulator of T-cell responses (17–19). Building on this knowledge, we postulated that SST might exert its influence on T cells by modulating GSK3 through the SSTR3 pathway, consequently impacting T-cell immune responses.

**Figure 6:**
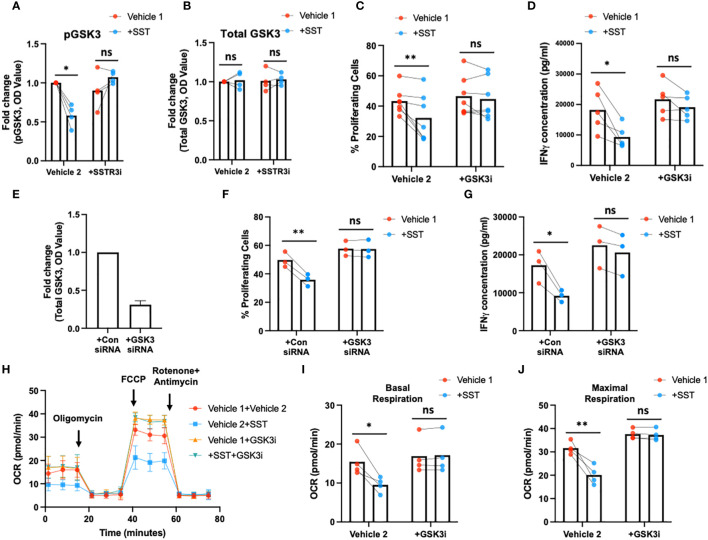
SST modulates T-cell responses through GSK3. **(A, B)** Human purified T cells were pre-treated with SSTR3 inhibitor (SSTRi, MK-4256, 2nM) for 30min. Cells were then incubated with SST (100nM) for 30min. The phosphorylation of GSK3 (inactive form) and total GSK3 are measured using ELISA. SST decreases the phosphorylation of GSK3 (release the active form) **(A)**, n=4) without change total GSK3 expression **(B)**, n=4), which can be blocked by the inhibition of SSTR3 **(A)**. **(C, D)** Human purified T cells were labelled with CFSE and were pre-treated with GSK3 inhibitor (GSK3i, CHIR 99021, 20nM) for 30min. Cells were then incubated with SST (100nM) for 30min. After that, T-cell activation beads were used to stimulate T cells at 1:2 ratio (beads: cells) for 4 days. T-cell proliferation were measured by the dilution of CFSE and quantified by flow cytometry. IFNγ from T cells was measured by ELISA. The blockade of GSK3 inhibits the effect of SST on T-cell proliferation **(C)**, n=7) and IFNγ production **(D)**, n=5). **(E-G)** CFSE labeled human primary T cells were transfected with either control siRNA or GSK3beta siRNA mix. On day 2, the expression of GSK3 were measured by ELISA **(E)**. After transfection, cells were pre-treated with SST for 30min and were stimulated with T-cell activation beads (αCD2, αCD3 and αCD28) for 4 days. CFSE dilution and IFNg expression was measured by flow cytometry and ELISA respectively (n=3). **(H-J)** Human purified T cells were pre-treated with GSK3 inhibitor (GSK3i, CHIR 99021, 20nM) for 30min. Cells were then incubated with SST (100nM) for 30min. After that, T-cell activation beads were used to stimulate T cells at 1:2 ratio (beads: cells) for 24hrs. XF Mitochondria stress test was used to detect T-cell mitochondrial respiration. The blockade of GSK3 reverses the effect of SST on T-cell mitochondrial respiration **(H-J)**, n=4). *Vehicle 1:* ddH_2_O. *Vehicle 2:* DMSO. Data is presented as individual dots or mean±SD. Repeat measure two-way ANOVA, Two-stage step-up method of Benjamini, Krieger and Yekutieli to correct for multiple comparison by controlling the False Discovery Rate. ns, not significant, *p<0.05 and **p<0.01.

To test that hypothesis, we first measured pGSK3 (Ser9, *leading to GSK3 inactivation*) and total GSK3 using ELISA (**Figures 6A, B**). Treatment of SST significantly decreases pGSK3 without affecting total GSK3, which could be blocked by SSTR3i (**Figures 6A, B**). Unlike other kinases, phosphorylation of GSK3 leads to inactivation of the enzyme (20), indicating SST enhances GSK3 function through SSTR3. Furthermore, we showed that the inhibition of GSK3 could rescue the effect of SST on T-cell proliferation, IFNγ expression (**Figures 6C–G**) and mitochondrial respiration (**Figures 6H–J**). These data together suggested that SST down-regulates T-cell responses and metabolism through SSTR3-GSK3 pathway.

## Discussion

In this investigation, we studied the molecular mechanism underlying the immune regulatory role of SST on T cells. Building upon prior research, we demonstrated that SST exerts a direct influence in mitigating T-cell responses. This suppressive impact of SST is associated with reduction in T-cell mitochondrial respiration, while leaving T-cell glycolysis unaffected. Furthermore, our exploration unveiled that SST orchestrates the modulation of T-cell responses and metabolism through somatostatin receptor 3 (SSTR3) mediated activation of glycogen synthase kinase 3 (GSK3).

Under normal physiological conditions, SST is mainly produced in the digestive and nervous systems (1). with its production in the brain following a circadian rhythm (21). Aberrant elevations of SST are evident in conditions such as somatostatinoma and inflammatory bowel diseases (22, 23). As an inhibitory hormone, SST wields control over a spectrum of hormones, including growth hormone and gastrointestinal hormones (2). Because of that, therapeutic applications of SST extend to diverse conditions, including the treatment of disorders like acromegaly, gastrointestinal bleeding, and neuroendocrine tumors (24).

The immune regulatory effect of SST has been extensively studied. Early studies showed that SST and its analogues can down-regulates immune-cell activation, proliferation, and cytokine production (8, 10–12, 25, 26). Yet, the underlying molecular intricacies have, until now, remained somewhat elusive. Our study sought to bridge this gap by elucidating that SST selectively targets oxidative phosphorylation (OXPHOS) in T cells, thereby impacting their effector function without a concurrent reduction in glycolysis. Recent studies have shown that cellular metabolism is essential for regulating effector functions of T cells (27). Upon stimulation through T-cell receptor (TCR), T cells undergo rapid metabolic transition from oxidative phosphorylation (OXPHOS) to glycolysis (28), which is important for the effector function of T cells during early phase of activation (28–30). Despite the switch from OXPHOS to glycolysis during T-cell activation, OXPHOS still works as an important contributor to provide complementary ATP (31) and OXPHOS associated metabolites are also crucial for effector function of T cells (32, 33). We observed that SST preferentially targeting OXPHOS but spare T-cell glycolysis, which may explain why SST may only partially but not completely reduce T-cell responses. Importantly, SST does not appear to directly influence T-cell receptor (TCR) signaling pathways; instead, it exerts its effects on CD28-mediated interleukin-2 (IL-2) production and GSK3 phosphorylation, suggesting a pivotal role for SST in shaping how T cells interact with antigen-presenting cells.

Regulatory T cells are important checkpoint to control autoimmunity and avoid exacerbated immune reactions. In this study, we used pan-T cells that includes both effector T cells and regulatory T cells. Therefore, it is possible that SST may regulate effector T-cell responses indirectly through enhance Treg function. While it is conceivable that SST may indirectly regulate effector T-cell responses by enhancing regulatory T-cell function, recent research implies that SST analogs, such as octreotide, may impede regulatory T-cell function in patients with neuroendocrine tumors by downregulating the expression of immune regulatory molecules, such as PD1 and CTLA4) (34). This underscores a dual impact of SST, influencing both non-regulatory and regulatory T-cell responses.

Glycogen synthase kinase 3 (GSK3), acknowledged as a negative regulator of T-cell responses (17–19, 35–37), exerts control over T-cell function by inhibiting T-bet, a pivotal transcriptional factor for type 1 T cells (Th1 and Tc1) (18, 35). The impact of GSK3 on human T cells has not been well studied. Our data suggested that SST signals through SSTR3 to activate GSK3 in human T cells and display relatively stronger impact on IFNγ, a signature cytokine for type 1 T cells. GSK3 is also a crucial molecule that controls multiple metabolic processes (38–41). Remarkably, GSK3 emerges as a potential metabolic checkpoint for T cells, mirroring its role in maintaining B cells in a quiescent state (41) — a regulatory process finely tuned by SST-SSTR3 interactions.

Taken together, these findings strongly suggest that SST exerts a down-regulatory effect on T-cell responses and metabolism through the SSTR3-GSK3 pathway. This unveiled signaling cascade sheds light on the intricate molecular mechanisms by which somatostatin orchestrates its regulatory influence on T-cell behavior, offering a deeper understanding of the interplay between SST-SSTR3-GSK3 and T-cell functions as well as providing experimental basis for future exploring therapeutic role of SST in the context of inflammatory diseases.

## Data availability statement

The original contributions presented in the study are included in the article/**Supplementary Material**. Further inquiries can be directed to the corresponding authors.

## Ethics statement

The studies involving humans were approved by Fujian Medical University ethics review board. The studies were conducted in accordance with the local legislation and institutional requirements. The participants provided their written informed consent to participate in this study.

## Author contributions

BZ: Conceptualization, Data curation, Formal Analysis, Investigation, Methodology, Project administration, Validation, Visualization, Writing – original draft, Writing – review & editing. HF: Data curation, Methodology, Writing – review & editing. HL: Data curation, Methodology. RL: Conceptualization, Funding acquisition, Supervision, Writing – original draft, Writing – review & editing.

## Supplementary material

Supplementary Figure 1 SST does not influence T-cell survival and TCR signaling pathway. Purified human primary T cells were treated either with vehicle or SST for 30min and then stimulated with T-cell activation beads (αCD2, αCD3 and αCD28) for 4 days. T-cell survival was measured by Live/Dead Aqua staining. Compared to vehicle, SST does not apparently change human T-cell survival. (n=5)

Supplementary Figure 2 SSTRs expression on human T cells. **(A)** Purified human primary T cells were isolated from peripheral blood using negative isolation kit. The expression of somatostatin receptors was measured by real-time PCR. Delta CT were calculated based on β-actin. sstr3 is predominant somatostatin receptor expressed by human peripheral T cells. (n=4). **(B)** Expression of SSTR3 by different CD4^+^ T-cell subsets (n=3). **(C)** Expression of SSTR3 by different CD8^+^ T-cell subsets (n=3). **(D)** Purified human primary T cells were either unstimulated or stimulated with T-cell activation beads (αCD2, αCD3 and αCD28) for 6hrs, 12hrs, 24hrs, 48hrs or 72hrs. SSTR3 expression were measured by real-time PCR. **(E)** Purified human primary T cells were either unstimulated or stimulated with T-cell activation beads (αCD2, αCD3 and αCD28) for 12hrs or 72hrs. SSTR3 expression were measured by flow cytometry. Nil, unstimulated T cells; Act, anti-CD2, CD3 and CD28 microbeads.
