# Supplementary material for: Somatostatin-SSTR3-GSK3 modulates human T-cell responses by inhibiting OXPHOS
Source: Front Immunol. 2024 Feb 15;15:1322670. doi: 10.3389/fimmu.2024.1322670 (PMC10902055; doi:10.3389/fimmu.2024.1322670)

Supplementary Figure 1. SST does not influence T-cell survival and TCR signaling pathway

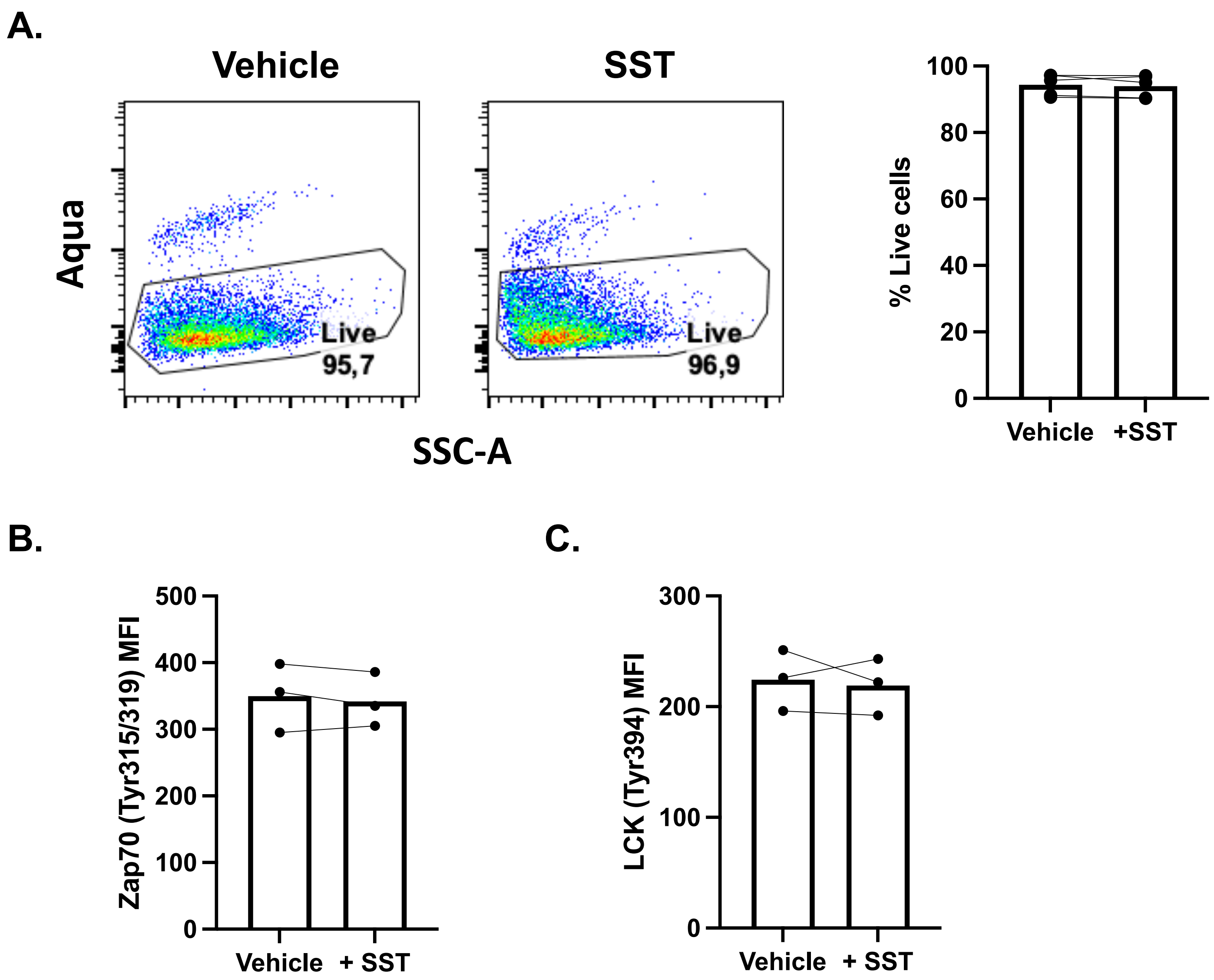

Supplementary Figure 2. SSTRs expression on human T cells

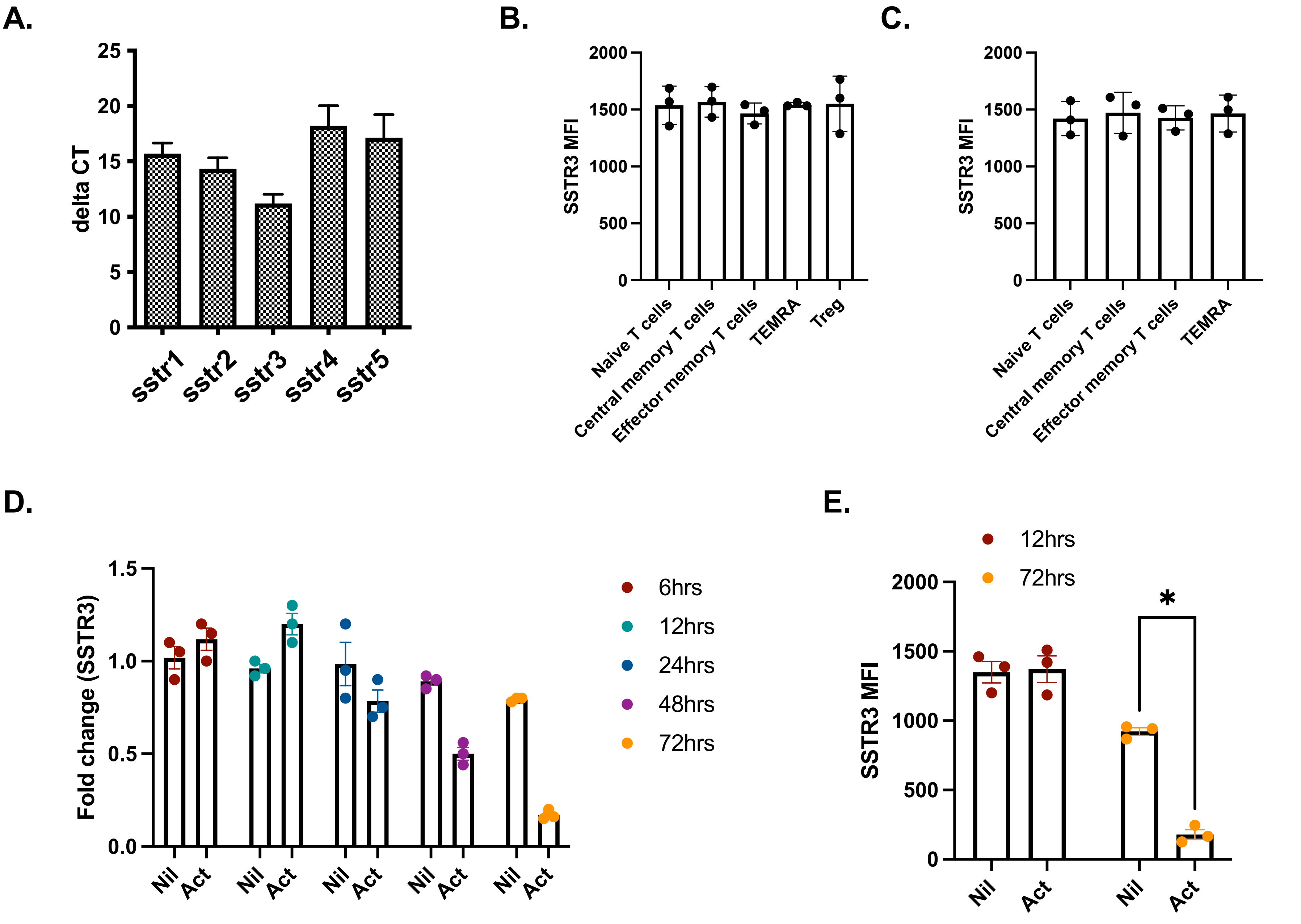

Supplement: Supplementary Figure 1 — SST does not influence T-cell survival and TCR signaling pathway. Purified human primary T cells were treated either with vehicle or SST for 30min and then stimulated with T-cell activation beads (αCD2, αCD3 and αCD28) for 4 days. T-cell survival was measured by Live/Dead Aqua staining. Compared to vehicle, SST does not apparently change human T-cell survival. (n=5) [file DataSheet_1.pdf]
